# Supplementary material for: A citric acid cycle-deficient Escherichia coli as an efficient chassis for aerobic fermentations
Source: Nat Commun. 2024 Mar 15;15:2372. doi: 10.1038/s41467-024-46655-4 (PMC10943122; doi:10.1038/s41467-024-46655-4)
Supplement: Supplementary file 1 — Supplementary Information [file 41467_2024_46655_MOESM1_ESM.doc]

**A citric acid cycle-deficient *Escherichia coli* as an efficient chassis for aerobic fermentations**

Zhou *et al.*


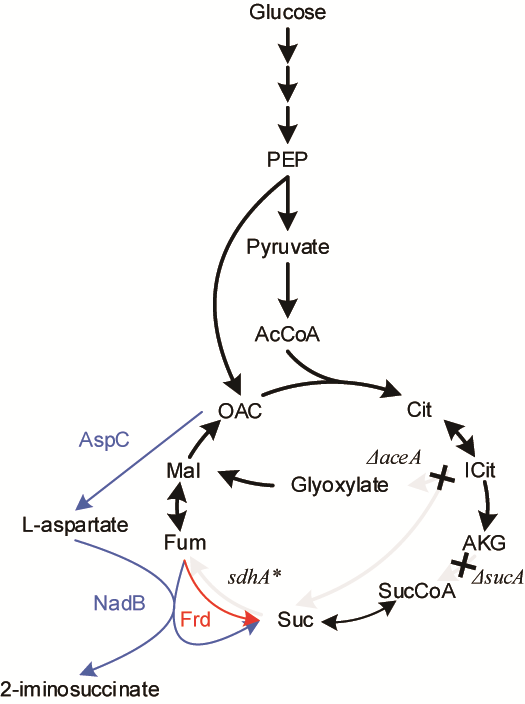


**Supplementary Figure 1.** Potential replenishment pathway for succinate through L-aspartate oxidase (NadB) and fumarate reductase (Frd) reactions ^1-3^.


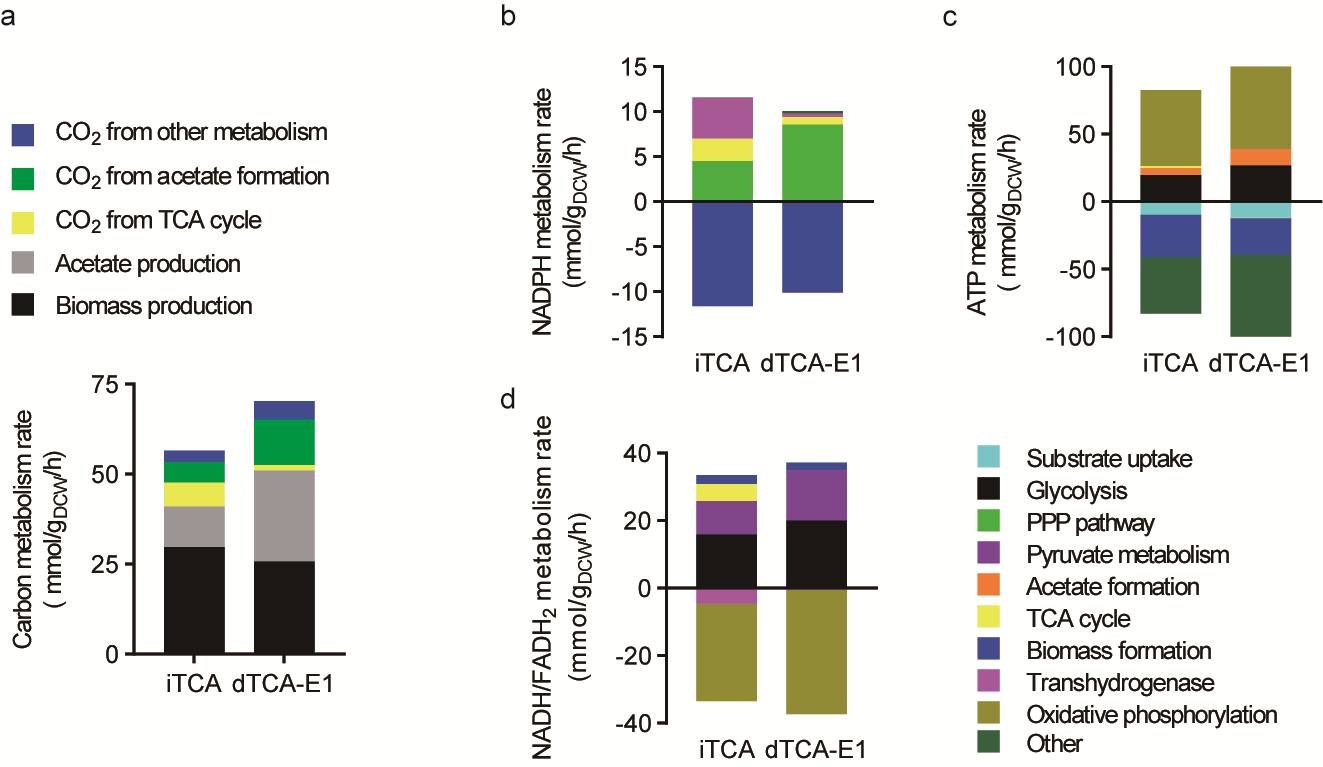


**Supplementary Figure 2.** Carbon and cofactors balance analysis of the evolved strain dTCA-E1 and the unevolved strain iTCA according to absolute metabolism rate (mmol/g_DCW_/h). **a** Carbon balance analysis of dTCA-E1 and iTCA. Values are means ± SD (n = 3 biological replicates). **b** NADPH metabolism balance analysis of dTCA-E1 and iTCA. Values are means ± SD (n = 3 biological replicates). **c** ATP metabolism balance analysis of dTCA-E1 and iTCA. Values are means ± SD (n = 3 biological replicates). **d** NADH/FADH_2_ metabolism balance analysis of dTCA-E1 and iTCA. Values are means ± SD (n = 3 biological replicates). Co-factor metabolism balance analysis was shown as the production rate (positive value) and consumption rate (negative value). dTCA-E1: evolved dTCA strain (Figure 1, Supplementary Table 1); iTCA: strain BW25113 *ΔpoxB*::*acs* with an intact TCA cycle (Figure 1, Supplementary Table 1). Source data are provided in Source Data file.

**
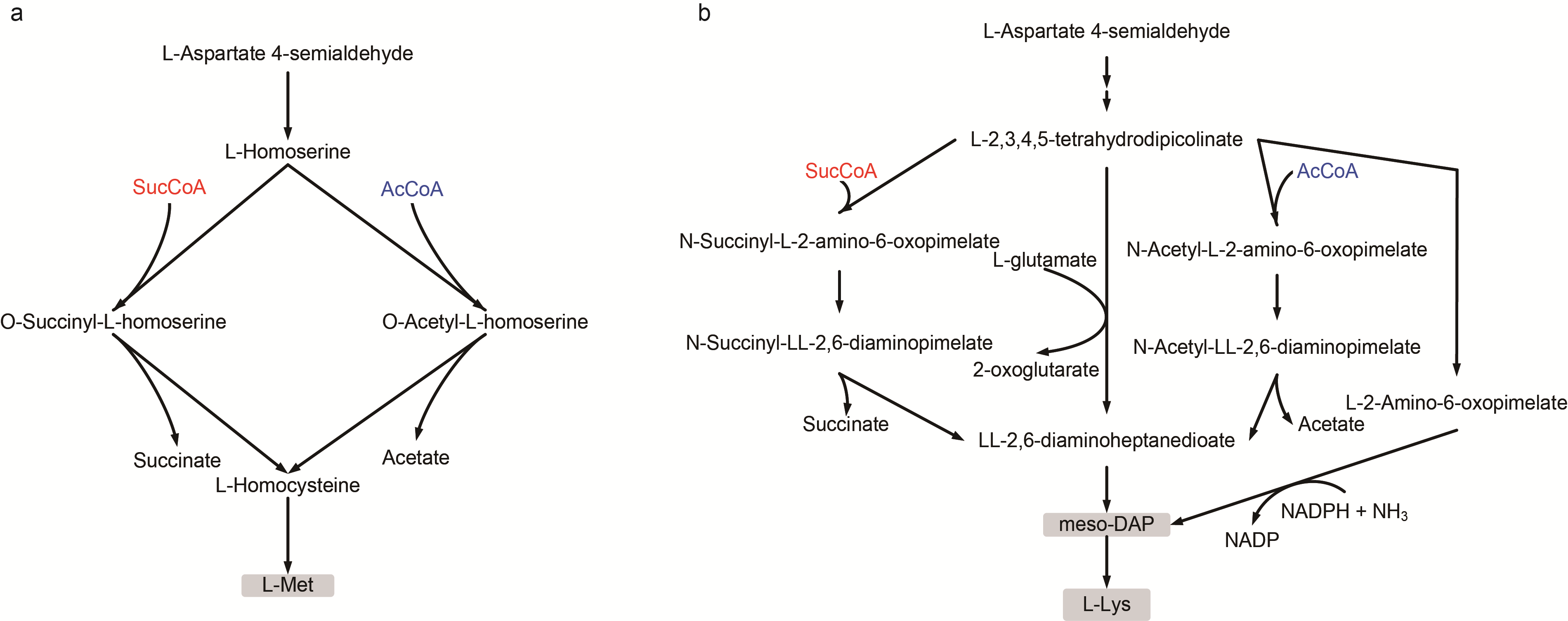
**

**Supplementary Figure 3.** Alternative pathways for synthesis of L-methionine (L-Met), meso-2,6-diaminoheptanedioate (meso-DAP) and L-lysine (L-Lys). **a** Alternative pathways for L-Met synthesis.^4^ **b** Alternative pathways for synthesis of meso-DAP and L-Lys.^5, 6^


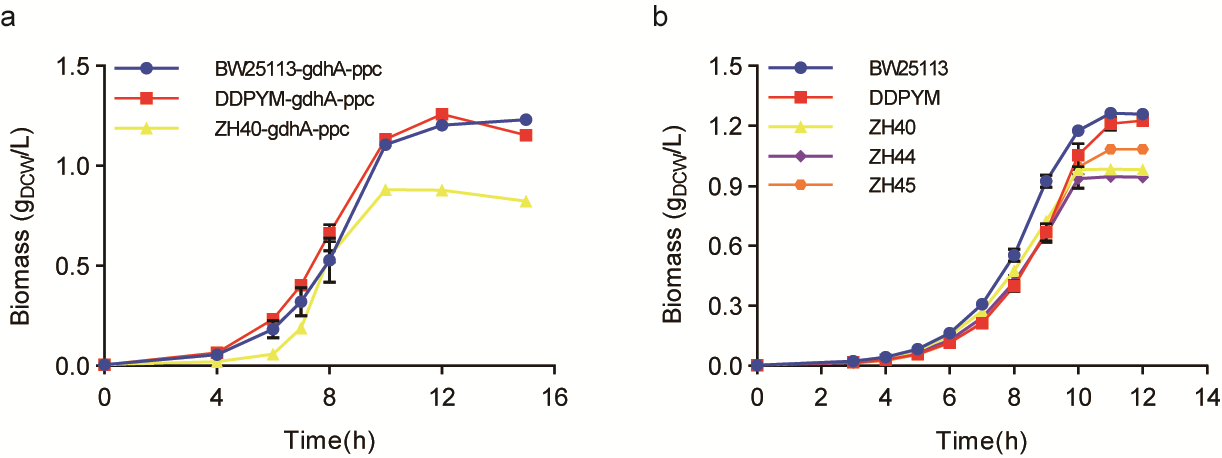


**Supplementary Figure 4.** Aerobic growth of the engineered strains in glucose minimal medium. **a** Aerobic growth of strains producing glutamate in glucose minimal medium. Values are means ± SD (n = 3 biological replicates). **b** Aerobic growth of strains producing acetate in glucose minimal medium. Values are means ± SD (n = 3 biological replicates). Source data are provided as a Source Data file. Details of all strains were listed in Supplementary Table 1.


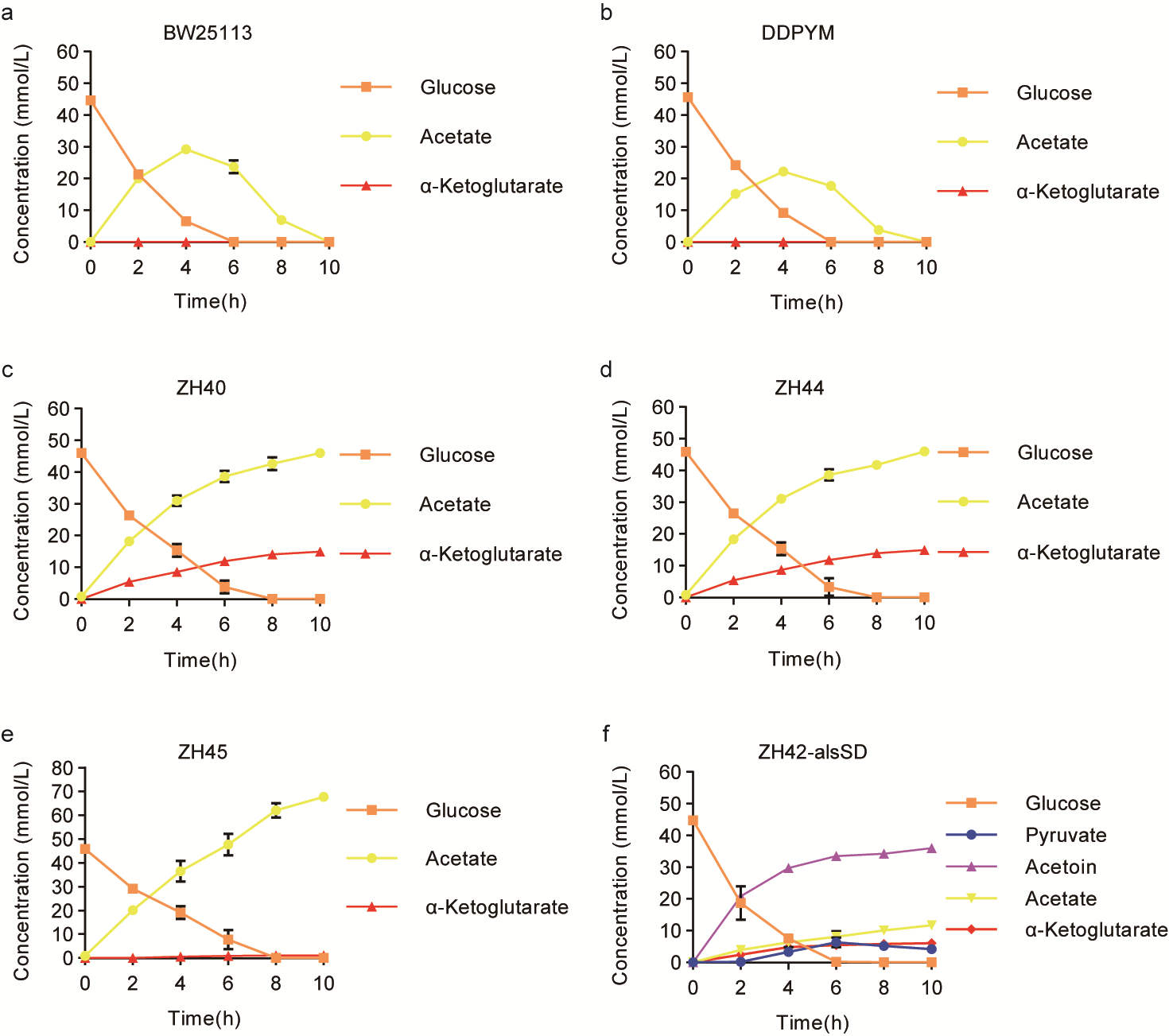


**Supplementary Figure 5.** Glucose catabolism of TCA cycle-deficient *E. coli* strains comparing to TCA cycle intact strains during whole-cell biocatalysis. **a** Glucose catabolism of BW25113 during whole-cell biocatalysis. Values are means ± SD (n = 3 biological replicates). **b** Glucose catabolism of DDPYM during whole-cell biocatalysis. Values are means ± SD (n = 3 biological replicates). **c** Glucose catabolism of ZH40 during whole-cell biocatalysis. Values are means ± SD (n = 3 biological replicates). **d** Glucose catabolism of ZH44 during whole-cell biocatalysis. Values are means ± SD (n = 3 biological replicates). **e** Glucose catabolism of ZH45 during whole-cell biocatalysis. Values are means ± SD (n = 3 biological replicates). **f** Glucose catabolism of ZH42-alsSD during whole-cell biocatalysis. Values are means ± SD (n = 3 biological replicates). Source data are provided as a Source Data file. Details of all strains were listed in Supplementary Table 1.

**Supplementary Table 1.** Strains and plasmids used in this study

| Plasmids/Strains | Genotype | Source |
| --- | --- | --- |
| Strains |  |  |
| BW25113 | *E. coli* K12 *rrnB_T14_ΔlacZ_WJ16_hsdR514ΔaraBAD_AH33_ΔrhaBAD_LD78_* | 7 |
| dTCA | BW25113 *ΔaceAΔsucAΔgadAΔgadBΔpoxB*::*acs* | This work |
| iTCA | BW25113 *ΔpoxB*::*acs* | 8 |
| dTCA-E1 | Evolved dTCA | This work |
| dTCA-E2 | Evolved dTCA | This work |
| dTCA-E3 | Evolved dTCA | This work |
| dTCA-E4 | Evolved dTCA | This work |
| DDPYM | BW25113 *ΔdapD*::*dapH(Bs)-dapL(Bs)-patA(Bs) ΔmetA*::*yjcI(Bs)-metA(Bs)* | This work |
| ZH40 | DDPYM *ΔaceAΔsucABCD* | This work |
| ZH42 | ZH40 *ΔpoxBΔpta gltA(T109P)* | This work |
| ZH44 | ZH40 *ΔpoxB* | This work |
| ZH45 | ZH44 *gltA(T109P)* | This work |
| BW25113-gdhA-ppc | BW25113 with plasmid pSC2s-gdhA-ppc | This work |
| DDPYM-gdhA-ppc | DDPYM with plasmid pSC2s-gdhA-ppc | This work |
| ZH40-gdhA-ppc | ZH40 with plasmid pSC2s-gdhA-ppc | This work |
| ZH42-alsSD | ZH42 *ΔldhA*::P*tac-alsSD*; AlsS and AlsD are expressed under P*tac*. | This work |
| Plasmids |  |  |
| pET28b(+)-DAOCS | pMB1; Kan^+^; DAOCS is expressed under T7 promoter. | This work |
| pSC2s | pSC101; Str^+^; P*tac* promoter. | Our lab |
| pSC2s-gdhA-ppc | pSC101; Str^+^; *gdhA* and *ppc* are expressed under P*tac*. | This work |

**Supplementary Table 2.** Aerobic growth characteristics of unevolved TCA cycle-defective *E. coli* dTCA in glucose minimal medium after introducing mutations in succinate dehydrogenase and citrate synthase.^a^

|  | Specific growth rate  (h^-1^) | Biomass yield (g_DCW_/g) | Acetate yield (mol/mol) |
| --- | --- | --- | --- |
| dTCA *ΔsdhA* | 0.35±0.00 | 0.25±0.02 | 0.59±0.04 |
| dTCA *ΔsdhA glt(T109P*) | 0.44±0.00 (*p*=0.0001) | 0.28±0.01 (*p*=0.0639) | 1.01±0.06 (*p*=0.0009) |
| dTCA *ΔsdhA gltA(L10I,T109P)* | 0.44±0.00 (*p*=0.0001) | 0.29±0.03 (*p*=0.0834) | 1.01±0.08 (*p*=0.0033) |
| dTCA *ΔsdhA gltA(H157Y)* | 0.44±0.01 (*p*=0.0002) | 0.27±0.01 (*p*=0.2860) | 0.92±0.01 (*p*=0.0032) |
| dTCA *ΔsdhA gltA(I375T)* | 0.45±0.00 (*p*=2.17x10^-5^) | 0.27±0.01 (*p*=0.2061) | 0.97±0.03 (*p*=0.0004) |

^a^ Values are means ± SD (n = 3 biological replicates). Significant difference analysis was conducted comparing to dTCA *ΔsdhA*. All above *P* values were calculated by unpaired two-tailed *t*-test. Source data are provided as a Source Data file. Details of all strains were listed in Supplementary Table 1.

**Supplementary Table 3.** Growth characteristics of iTCA strain and dTCA-E1 strain in glucose minimal medium which were cultivated for ^13^C-metabolic flux analysis. ^a^

|  | iTCA | dTCA-E1 |
| --- | --- | --- |
| Specific growth rate (h^-1^) | 0.72 ± 0.01 | 0.62 ± 0.01 |
| Biomass yield (g_DCW_/g) | 0.42 ± 0.01 | 0.30 ± 0.01 |
| Acetate yield (mol/mol) | 0.61 ± 0.04 | 1.12 ± 0.01 |
| Glucose uptake rate (mmol/g_DCW_/h) | 9.4 ± 0.2 | 11.7 ± 0.3 |

^a^ Values are means ± SD (n = 3 biological replicates).

**Supplementary Table 4.** Mutation analysis of citrate synthase and succinate dehydrogenase in dTCA clones from ALE-1 culture that survived on glucose M9 medium plate. ^a^

| Clones | GltA | SdhC | SdhD | SdhA | SdhB |
| --- | --- | --- | --- | --- | --- |
| 1 |  |  |  | E 62 D |  |
| 2 |  |  |  | L 369 F |  |
| 3 |  |  | F 51 FS |  |  |
| 4 | H 130 Y |  |  | D 6 Y |  |
| 5 |  |  |  | T 522 M |  |
| 6 |  |  |  | E 305 K |  |
| 7 |  |  |  |  | Q 126 K |
| 8 |  |  |  | A 34 T, S 536 F |  |
| 9 |  |  |  | E 62 D, G 416 D, L 42 P |  |
| 10 |  |  |  | G 51 S |  |

^a^ FS: Frame shift.

The populations from the ALE-1 group of evolutionary experiments were streaked onto glucose M9 medium plates after the second transfer in M9 medium supplemented with 0.5 mL Luria-Bertani. Single clones that survived on glucose M9 plates were then randomly selected for PCR reactions and Sanger sequencing.

**Supplementary Table 5.** Mutation analysis of citrate synthase and succinate dehydrogenase in dTCA clones from ALE-2 culture that survived on glucose M9 medium plate. ^a^

| Clones | GltA | SdhC | SdhD | SdhA | SdhB |
| --- | --- | --- | --- | --- | --- |
| 1 |  |  |  | L 408 P |  |
| 2 |  |  |  | G 51 S |  |
| 3 |  |  |  | L 408 P |  |
| 4 |  |  |  | P 222 FS |  |
| 5 |  |  |  | P 222 FS |  |
| 6 |  |  |  | C 257 Y |  |
| 7 |  |  |  | T 134 I |  |
| 8 |  |  |  | G 51 S |  |
| 9 |  |  |  | D 287 FS |  |
| 10 |  |  |  | G 51 S |  |

^a^ FS: Frame shift.

The populations from the ALE-2 group of evolutionary experiments were streaked onto glucose M9 medium plates after the second transfer in M9 medium supplemented with 0.5 mL Luria-Bertani. Single clones that survived on glucose M9 plates were then randomly selected for PCR reactions and Sanger sequencing.

**Supplementary Table 6.** Mutation analysis of citrate synthase and succinate dehydrogenase in BW25113 *ΔaceAΔsucA* clones that survived on glucose M9 medium plate. ^a^

| Clones | GltA | SdhC | SdhD | SdhA | SdhB |
| --- | --- | --- | --- | --- | --- |
| 1 |  |  |  | L 73 FS |  |
| 2 |  |  | A 135 FS |  |  |
| 3 |  | *sdhD::insB1* |  |  |  |
| 4 |  |  | S 529 Y |  |  |
| 5 | K 7 FS |  |  |  | K 7 FS |
| 6 |  |  |  | L 73 FS |  |
| 7 |  |  |  | Q 135 Stop |  |
| 8 |  |  |  | L 73 FS |  |
| 9 |  |  | E 237 Stop |  |  |
| 10 |  |  | G 103 D |  |  |

^a^ FS: Frame shift; *insB1*: insertion sequence IS1 protein InsB; Stop: stop codon.

BW25113 *ΔaceAΔsucA*, which was pre-cultivated in Luria-Bertani medium, was washed twice with M9 medium and then spread on a glucose M9 medium plate. Single clones that survived on glucose M9 plates were randomly selected for PCR and Sanger sequencing.

**Supplementary Table 7.** Specific growth rate of strains with defective TCA cycle that grow in glucose minimal medium with supplements or pathway engineering.^a^

| **Strain** | Specific growth rate (h^-1^) |
| --- | --- |
| BW25113 *ΔaceAΔsucA* | NA ^b^ |
| BW25113 *ΔaceAΔsucAΔsdhA* | 0.32±0.04 |
| BW25113 *ΔaceAΔsucA* (with 2 mmol/L Suc) | 0.52±0.05 (*p*=3.51x10^-5^) |
| BW25113 *ΔaceAΔsucA* (with 3AAs) | 0.51±0.07 (*p*=4.38x10^-4^) |
| BW25113 | 0.54±0.06 (*p*=2.12x10^-5^) |
| DDPYM | 0.54±0.05 (*p*=6.66x10^-6^) |
| DDPYM *ΔaceAΔsucA* | 0.51±0.05 (*p*=4.20x10^-5^) |
| DDPYM *ΔaceAΔsucABCD* | 0.52±0.04 (*p*=3.39x10^-6^) |

^a^ Aerobic growth was conducted in 96-well plates shaking under 800 rpm and 37 °C. Suc: 2 mmol/L succinate. 3AAs: 1 mmol/L for each of meso-2,6-diaminoheptanedioate (meso-DAP), lysine and methionine. Values are means ± SD (n = 6 biological replicates). Significant difference analysis was conducted comparing to BW25113 *ΔaceAΔsucAΔsdhA*. All above *P* values were calculated by unpaired two-tailed *t*-test. Source data are provided as a Source Data file. Details of all strains were listed in Supplementary Table 1.

^b^ BW25113 *ΔaceAΔsucA* could not grow in glucose minimal medium*.*

**Supplementary Table 8.** Aerobic growth and acetate production of *E. coli* BW25113 and the engineered strains in glucose minimal medium.^a^

|  | BW25113 | DDPYM | ZH40 | ZH44 | ZH45 |
| --- | --- | --- | --- | --- | --- |
| *μ* (h^-1^) | 0.66±0.01 | 0.63±0.00 | 0.63±0.01 | 0.64±0.00 | 0.69±0.00 |
| *Y_x/s_* (g_DCW_/g_glc_) | 0.36±0.03 | 0.33±0.01 | 0.28±0.02 | 0.27±0.02 | 0.32±0.01 |
| *q_glucose_* (mmol/g_DCW_/h) | 10.28±0.67 | 10.50±0.49 | 12.58±0.79 | 13.25±0.79 | 11.98±0.20 |
| *r_acetate_* (mmol/g_DCW_/h) | 5.51±0.08 | 4.88±0.41 | 7.12±0.40 | 6.94±0.53 | 7.99±0.25 |
| production *_acetate_* (mmol/g_DCW_) | 8.41±0.20 | 7.79±0.64 | 11.23±0.63 | 10.77±0.84 | 11.66±0.37 |
| *Y_acetate/glucose_* (mol/mol)_growth_ | 0.54±0.04 | 0.53±0.03 | 0.61±0.06 | 0.63±0.05 | 0.71±0.12 |

^a^ Values are means ± SD (n = 3 biological replicates). Source data are provided in Source Data file. Details of all strains were listed in Supplementary Table 1.

**Supplementary Note 1：Metabolic network model of *E. coli* used for ^13^C metabolic flux analysis**

| **Glycolysis** | |
| --- | --- |
| v_1_ | Gluc.ext (abcdef) + PEP (ghi) → G6P (abcdef) + Pyr (ghi) |
| v_2_ | G6P (abcdef) ↔ F6P (abcdef) |
| v_3_ | F6P (abcdef) + ATP ↔ FBP (abcdef) |
| v_4_ | FBP (abcdef) ↔ DHAP (cba) + GAP (def) |
| v_5_ | DHAP (abc) ↔ GAP (abc) |
| v_6_ | GAP (abc) ↔ 3PG (abc) + ATP + NADH |
| v_7_ | 3PG (abc) ↔ PEP (abc) |
| v_8_ | PEP (abc) → Pyr (abc) + ATP |
|  | |
| **Pentose Phosphate Pathway** | |
| v_9_ | G6P (abcdef) → 6PG (abcdef) + NADPH |
| v_10_ | 6PG (abcdef) → Ru5P (bcdef) + CO_2_ (a) + NADPH |
| v_11_ | Ru5P (abcde) ↔ X5P (abcde) |
| v_12_ | Ru5P (abcde) ↔ R5P (abcde) |
| v_13_ | X5P (abcde) ↔ TK-C2 (ab) + GAP (cde) |
| v_14_ | F6P (abcdef) ↔ TK-C2 (ab) + E4P (cdef) |
| v_15_ | S7P (abcdefg) ↔ TK-C2 (ab) + R5P (cdefg) |
| v_16_ | F6P (abcdef) ↔ TA-C3 (abc) + GAP (def) |
| v_17_ | S7P (abcdefg) ↔ TA-C3 (abc) + E4P (defg) |
|  | |
| **Entner-Doudoroff Pathway** | |
| v_18_ | 6PG (abcdef) → KDPG (abcdef) |
| v_19_ | KDPG (abcdef) → Pyr (abc) + GAP (def) |
|  | |
| **TCA Cycle** | |
| v_20_ | Pyr (abc) → AcCoA (bc) + CO_2_ (a) + NADH |
| v_21_ | OAC (abcd) + AcCoA (ef) → Cit (dcbfea) |
| v_22_ | Cit (abcdef) ↔ ICit (abcdef) |
| v_23_ | ICit (abcdef) ↔ AKG (abcde) + CO_2_ (f) + NADPH |
| v_24_ | AKG (abcde) → SucCoA (bcde) + CO_2_ (a) + NADH |
| v_25_ | SucCoA (abcd) ↔ Suc (½ abcd + ½ dcba) + ATP |
| v_26_ | Suc (½ abcd + ½ dcba) ↔ Fum (½ abcd + ½ dcba) + FADH_2_ |
| v_27_ | Fum (½ abcd + ½ dcba) ↔ Mal (abcd) |
| v_28_ | Mal (abcd) ↔ OAC (abcd) + NADH |
|  | |
| **Glyoxylate Shunt** | |
| v_29_ | ICit (abcdef) ↔ Glyox (ab) + Suc (½ edcf + ½ fcde) |
| v_30_ | Glyox (ab) + AcCoA (cd) → Mal (abdc) |
|  | |
| **Amphibolic Reactions** | |
| v_31_ | Mal (abcd) → Pyr (abc) + CO_2_ (d) + NADPH |
| v_32_ | PEP (abc) + CO_2_ (d) → OAC (abcd) |
| v_33_ | OAC (abcd) + ATP → PEP (abc) + CO_2_ (d) |
|  | |
| **Acetic Acid Formation** | |
| v_34_ | AcCoA (ab) ↔ Ac (ab) + ATP |
|  | |
| **Amino Acid Biosynthesis** | |
| v_35_ | AKG (abcde) + NADPH + NH_3_ → Glu (abcde) |
| v_36_ | Glu (abcde) + ATP + NH_3_ → Gln (abcde) |
| v_37_ | Glu (abcde) + ATP + 2 NADPH → Pro (abcde) |
| v_38_ | Glu (abcde) + CO_2_ (f) + Gln (ghijk) + Asp (lmno) + AcCoA (pq) + 5 ATP + NADPH → Arg (abcdef) + AKG (ghijk) + Fum (lmno) + Ac (pq) |
| v_39_ | OAC (abcd) + Glu (efghi) → Asp (abcd) + AKG (efghi) |
| v_40_ | Asp (abcd) + 2 ATP + NH3 → Asn (abcd) |
| v_41_ | Pyr (abc) + Glu (defgh) → Ala (abc) + AKG (defgh) |
| v_42_ | 3PG (abc) + Glu (defgh) → Ser (abc) + AKG (defgh) + NADH |
| v_43_ | Ser (abc) ↔ Gly (ab) + MEETHF (c) |
| v_44_ | Gly (ab) ↔ CO_2_ (a) + MEETHF (b) + NADH + NH3 |
| v_45_ | Thr (abcd) → Gly (ab) + AcCoA (cd) + NADH |
| v_46_ | Ser (abc) + AcCoA (de) + 3 ATP + 4 NADPH + SO_4_ → Cys (abc) + Ac (de) |
| v_47_ | Asp (abcd) + Pyr (efg) + Glu (hijkl) + SucCoA (mnop) + ATP + 2 NADPH →  LL-DAP (½ abcdgfe + ½ efgdcba) + AKG (hijkl) + Suc (½ mnop + ½ ponm) |
| v_48_ | LL-DAP (½ abcdefg + ½ gfedcba) → Lys (abcdef) + CO_2_ (g) |
| v_49_ | Asp (abcd) + 2 ATP + 2 NADPH → Thr (abcd) |
| v_50_ | Asp (abcd) + METHF (e) + Cys (fgh) + SucCoA (ijkl) + ATP + 2 NADPH →  Met (abcde) + Pyr (fgh) + Suc (½ ijkl + ½ lkji) + NH_3_ |
| v_51_ | Pyr (abc) + Pyr (def) + Glu (ghijk) + NADPH → Val (abcef) + CO_2_ (d) + AKG (ghijk) |
| v_52_ | AcCoA (ab) + Pyr (cde) + Pyr (fgh) + Glu (ijklm) + NADPH →  Leu (abdghe) + CO_2_ (c) + CO_2_ (f) + AKG (ijklm) + NADH |
| v_53_ | Thr (abcd) + Pyr (efg) + Glu (hijkl) + NADPH → Ile (abfcdg) + CO_2_ (e) + AKG (hijkl) + NH_3_ |
| v_54_ | PEP (abc) + PEP (def) + E4P (ghij) + Glu (klmno) + ATP + NADPH →  Phe (abcefghij) + CO_2_ (d) + AKG (klmno) |
| v_55_ | PEP (abc) + PEP (def) + E4P (ghij) + Glu (klmno) + ATP + NADPH →  Tyr (abcefghij) + CO_2_ (d) + AKG (klmno) + NADH |
| v_56_ | Ser (abc) + R5P (defgh) + PEP (ijk) + E4P (lmno) + PEP (pqr) + Gln (stuvw) + 3 ATP + NADPH →  Trp (abcedklmnoj) + CO_2_ (i) + GAP (fgh) + Pyr (pqr) + Glu (stuvw) |
| v_57_ | R5P (abcde) + FTHF (f) + Gln (ghijk) + Asp (lmno) + 5 ATP →  His (edcbaf) + AKG (ghijk) + Fum (lmno) + 2 NADH |
|  | |
| **One-Carbon Metabolism** | |
| v_58_ | MEETHF (a) + NADH → METHF (a) |
| v_59_ | MEETHF (a) → FTHF (a) + NADPH |
|  | |
| **Oxidative Phosphorylation** | |
| v_60_ | NADH + ½ O_2_ → 2 ATP |
| v_61_ | FADH2 + ½ O_2_ → 1 ATP |
|  | |
| **Transhydrogenation** | |
| v_62_ | NADH ↔ NADPH |
|  | |
| **ATP Hydrolysis** | |
| v_63_ | ATP → ATP:ext |
|  | |
| **Transport** | |
| v_64_ | Ac (ab) → Ac.ext (ab) |
| v_65_ | CO_2_ (a) → CO_2_.ext (a) |
| v_66_ | O_2_.ext → O_2_ |
| v_67_ | NH_3_.ext → NH_3_ |
| v_68_ | SO_4_.ext → SO_4_ |
|  | |
| **Biomass Formation** | |
| v_69_ | 0.470 Ala + 0.271 Arg + 0.236 Asn + 0.236 Asp + 0.084 Cys + 0.280 Glu + 0.280 Gln + 0.432 Gly + 0.082 His + 0.215 Ile + 0.377 Leu + 0.279 Lys + 0.107 Met + 0.153 Phe + 0.178 Pro + 0.232 Ser + 0.244 Thr + 0.052 Trp + 0.131 Tyr + 0.314 Val + 0.249 G6P + 0.071 F6P + 0.782 R5P + 0.082 GAP + 0.589 3PG + 0.051 PEP + 0.083 Pyr + 1.711 AcCoA + 0.087 AKG + 0.351 OAC + 0.459 MEETHF + 32.885 ATP + 3.941 NADPH → 36.332 Biomass + 1.458 NADH |
|  | |
| **CO_2_ Exchange** | |
| v_70_ | CO_2_.unlabeled (a) + CO_2_ (b) → CO_2_ (a) + CO_2_.out (b) |

The net effect of reaction v_70_ is exchange of intracellular CO_2_ for an unlabeled CO_2_ without affecting intracellular carbon balances.

**Supplementary references**

1. Hirsch, C.A., Davis, B.D., Rasminsky, M. & Lin, E.C.C. A Fumarate Reductase in *Escherichia Coli* Distinct from Succinate Dehydrogenase. *J Biol Chem.* **238**, 3770-3774 (1963).

2. Korshunov, S. & Imlay, J.A. Two sources of endogenous hydrogen peroxide in *Escherichia coli*. *Mol Microbiol.* **75**, 1389-1401 (2010).

3. Hermes, F.A. & Cronan, J.E. An NAD synthetic reaction bypasses the lipoate requirement for aerobic growth of *Escherichia coli* strains blocked in succinate catabolism. *Mol Microbiol.* **94**, 1134-1145 (2014).

4. Zubieta, C., Arkus, K.A.J., Cahoon, R.E. & Jez, J.M. A single amino acid change is responsible for evolution of acyltransferase specificity in bacterial methionine biosynthesis. *J Biol Chem.* **283**, 7561-7567 (2008).

5. Scapin, G. & Blanchard, J.S. Enzymology of bacterial lysine biosynthesis. *Adv Enzymol.* **72**, 279-324 (1998).

6. Born, T.L. & Blanchard, J.S. Structure/function studies on enzymes in the diaminopimelate pathway of bacterial cell wall biosynthesis. *Curr Opin Chem Biol.* **3**, 607-613 (1999).

7. Baba, T. *et al.* Construction of *Escherichia coli* K-12 in-frame, single-gene knockout mutants: the Keio collection. *Mol Syst Biol.* **2** (2006).

8. Lin, B. *et al.* Reconstitution of TCA cycle with DAOCS to engineer *Escherichia coli* into an efficient whole cell catalyst of penicillin G. *Proc Natl Acad Sci USA.* **112**, 9855-9859 (2015).
