# Supplementary material for: A citric acid cycle-deficient Escherichia coli as an efficient chassis for aerobic fermentations
Source: Nat Commun. 2024 Mar 15;15:2372. doi: 10.1038/s41467-024-46655-4 (PMC10943122; doi:10.1038/s41467-024-46655-4)
Supplement: Supplementary file 3 — Description of Additional Supplementary Files [file 41467_2024_46655_MOESM3_ESM.pdf]

Description of Additional Supplementary Files

File Name: Supplementary Data 1
Description: Gene mutations in dTCA strain before ALE

File Name: Supplementary Data 2
Description: Gene mutations in dTCA-E1 strain

File Name: Supplementary Data 3
Description: Gene mutations in dTCA-E2 strain

File Name: Supplementary Data 4
Description: Gene mutations in dTCA-E3 strain

File Name: Supplementary Data 5
Description: Gene mutations in dTCA-E4 strain

File Name: Supplementary Data 6
Description: KEGG modules with mutations common to four evolved strains (dTCA-E1, dTCA-E2, dTCA-E3, dTCA-E4)

File Name: Supplementary Data 7
Description: Mass isotopomer distributions from parallel labeling experiments

File Name: Supplementary Data 8
Description: Results of 13C-metabolic flux analysis and co-factor balances analysis

File Name: Supplementary Data 9
Description: Measure of intracellular succinyl-CoA level via LC-MS/MS

File Name: Supplementary Data 10
Description: Details for genome editing and plasmid construction.
